# Single Ventricularization Followed by Separation for Infected Ventricular Septal Rupture

**DOI:** 10.1016/j.atssr.2023.02.018

**Published:** 2023-03-05

**Authors:** Sho Takemoto, Hiromichi Sonoda, Yoshihisa Tanoue, Tomoki Ushijima, Akira Shiose

**Affiliations:** 1 Department of Cardiovascular Surgery, Kyushu University Hospital, Fukuoka, Japan

## Abstract

We report a total reconstruction of the interventricular septum for infected patches collapsing after repair of a postinfarction ventricular septal rupture (VSR). Patch infection developed after double-patch repair of a VSR in a 56-year-old man. He underwent extensive débridement of the patches and almost all the remaining interventricular septum, resulting in a single ventricle. We performed biventricular reconstruction using 2 patches, tricuspid valve replacement, and pacemaker implantation. Our method can provide reliable biventricular reconstruction even if it becomes a single ventricle by débridement. It can be helpful as a lifesaving operation to repair postinfarction VSR with fatal infection.

Postoperative patch infection after repair of ventricular septal rupture (VSR) is a catastrophic complication that often requires removal of the infected material and aggressive débridement of the surrounding tissues to overcome serious infection and subsequent hemodynamic collapse. Reconstruction of the débrided interventricular septum (IVS) depends on the extent of defective tissue. Furthermore, valve operation and permanent pacemaker implantation are required concomitantly if the mitral and tricuspid apparatus and atrioventricular node are involved. Herein, we describe an unprecedented case of biventricular repair for single ventricularization ascribable to débridement for methicillin-resistant *Staphylococcus aureus* (MRSA)–infected patches collapsing after repair of a postinfarction VSR. Our method enables the radical débridement and robust reconstruction essential for surmounting the fatal infection.

Inferoposterior acute myocardial infarction, followed by inferoposterior infarct VSR, led to cardiogenic shock in a 56-year-old man. After 9 days of cardiogenic shock treatment with an intra-aortic balloon pump (IABP) and venoarterial extracorporeal membrane oxygenation, the VSR was repaired by the double-patch method with 2 bovine pericardial patches through right anterior ventriculotomy^1^ (Figure 1A). The initial procedure was successful, but MRSA was isolated from the blood culture on the third postoperative day. Bloodstream infection was uncontrollable, regardless of multidrug antibiotic treatment. Concurrently, the patient’s hemodynamics rapidly collapsed, and the IABP was promptly reintroduced. Echocardiography revealed a left-to-right shunt through the collapsed VSR patch (Figure 1B) and massive vegetation on the right ventricular (RV) side of the VSR patch (Figure 1C).

**Figure 1:**
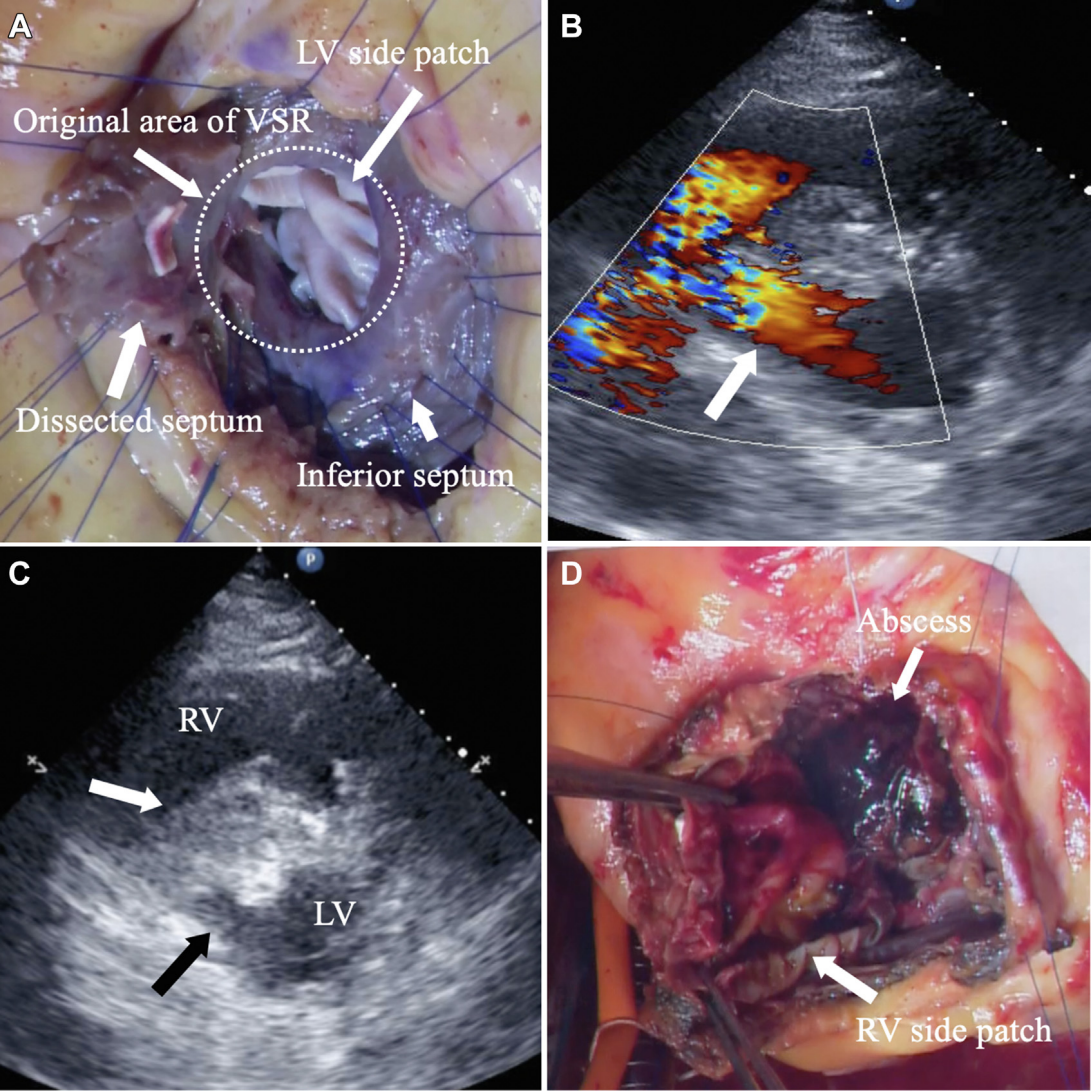
(A) Intraoperative photograph through right ventriculotomy at the initial operation shows the original area of ventricular septal rupture (VSR) and left ventricular (LV) side patch of double-patch repair. (B) Transthoracic color Doppler echocardiography shows relapse of ventricular septal rupture (arrow). (C) Transthoracic echocardiography shows large vegetation (white arrow) and collapse of the patch (black arrow). (D) Intraoperative image shows a large abscess (arrow) between the 2 patches. (RV, right ventricle.)

Because hemodynamics were exacerbated under IABP support, emergent redo operation was necessary. After establishment of cardiopulmonary bypass and induction of cardioplegic arrest, the previous right atriotomy and ventriculotomy procedures were reopened. Massive abscesses were found on the surface of the RV side of the VSR patch, and the infected hematoma filled the space between the 2 patches (Figure 1D). The remaining IVS was extensively infectious; mainly, the basal inferoposterior septum was necrotic except for the small part of the anterior septal rim. In addition, the infection also involved the anterior and septal portions of the tricuspid valve and annulus.

First, the basal inferoposterior septum and surrounding vulnerable and infectious tissues were thoroughly débrided, leaving only a small part of the IVS at the anterior and just below the aortic valve. The IVS at the inferoposterior portion was almost excised through the free wall, including the tricuspid valve. Most of the IVS was resected, resulting in an almost single ventricle. According to the extended sandwich technique, as previously described^2^ with minor modifications, double-patch repair with circularly trimmed bovine pericardial patches was repeated (Figures 2A, 2B). The left ventricular (LV) and RV side patch sizes were 80 × 90 mm and 90 × 90 mm, respectively. Eleven interrupted 3-0 polypropylene mattress sutures were applied at the edge of the defective IVS. Five of these sutures penetrated toward the outside of the free wall at the inferior margin to fix each side patch to sandwich the posterior descending artery. The sutures were reinforced with felt strips (Figures 2C, 2D).

**Figure 2:**
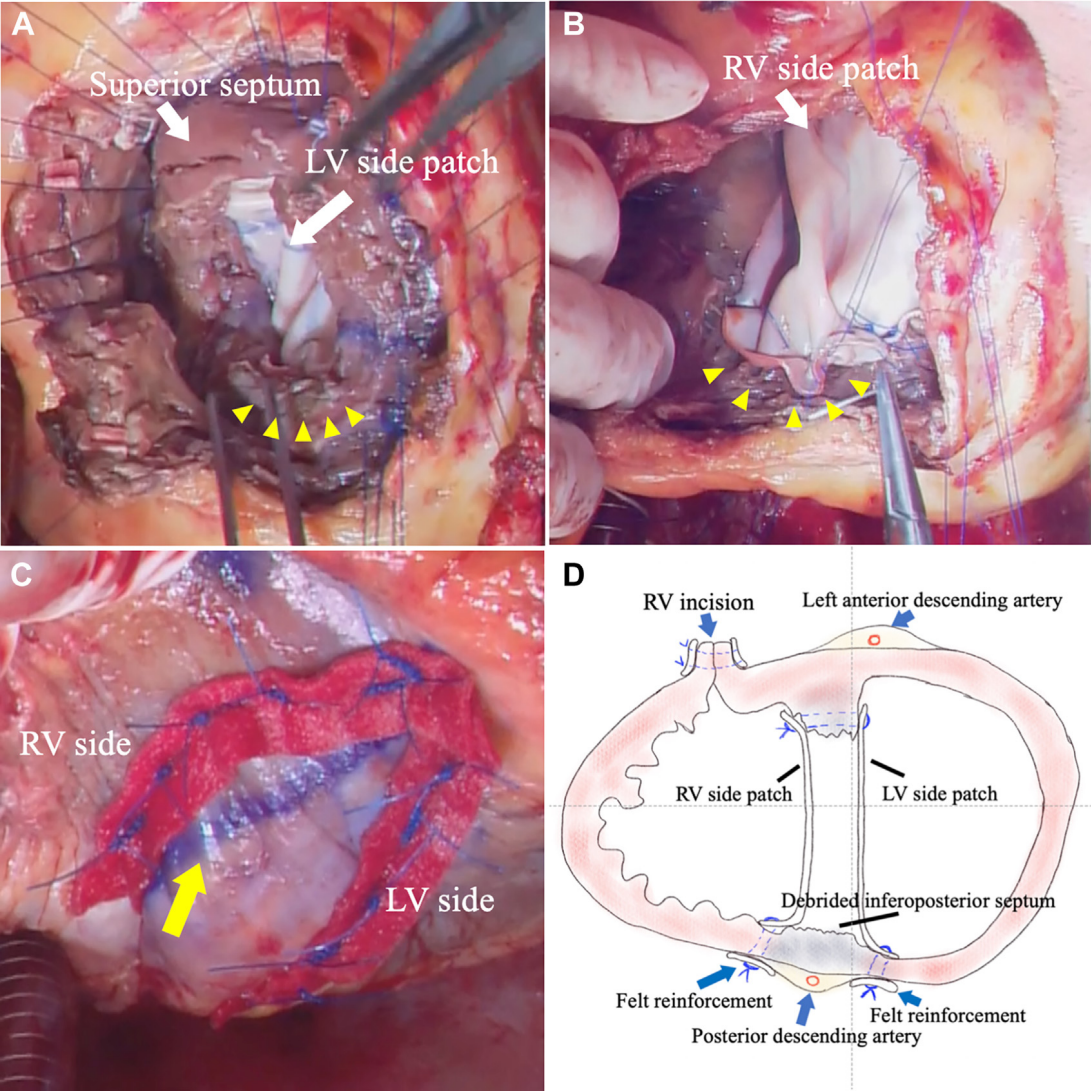
(A) Significant ventricular septal defect after extensive débridement. The arrowheads indicate the inferior ventricular septum, entirely débrided and reconstructed with penetrating mattress sutures toward the outside of the left ventricular (LV) wall. (B) Right ventricular (RV) side patch sutured with mattress sutures. (C) Felt reinforcements on both sides of the posterior descending artery. The arrow points to the purple line marker on the posterior descending artery. (D) Schematic diagram of repeated double-patch repair. Mattress sutures were applied through the left and right ventricular free walls on both sides of the posterior descending artery.

Second, tricuspid valve replacement was performed. A 25-mm Carpentier-Edwards Perimount Magna mitral valve bioprosthesis (Edwards Lifesciences) was implanted in the intra-annular position of the tricuspid valve. Finally, biventricular epicardial pacemaker leads were placed on the RV and LV surfaces, and cardiac resynchronization therapy was introduced for complete atrioventricular block and interventricular dyssynchrony.

The patient was successfully weaned off cardiopulmonary bypass with IABP support. He was treated with a 4-week multidrug antibiotic regimen (intravenous administration of vancomycin, daptomycin, and gentamicin and oral administration of rifampicin), followed by 4-week intravenous administration of vancomycin and lifelong oral administration of sulfamethoxazole-trimethoprim. He was discharged 3 months after surgery. Approximately 1.5 years after operation, the patient was doing well with no recurrence of infection, residual shunt, or tricuspid perivalvular leakage.

## Comment

The frequency of VSR as a mechanical complication after acute myocardial infarction is 0.35% among all cardiovascular surgeries, and its operative death is still as high as 24.8%.^3^ Reoperation for patch infection after VSR repair is rare, and operative mortality should increase further. In our case, the MRSA bacteremia may not have been cured during the initial operation. As a result, the VSR patch was infected postoperatively, and the suture line of the patch and the tricuspid annulus collapsed. The infected materials and tissues must be removed for infection control. However, total débridement of the IVS is considered a challenging strategy because reconstruction of the IVS is often technically difficult, and conduction disturbance and tricuspid valve dysfunction are unwanted complications. In this case, after removal of the infected patch and thorough débridement of the surrounding tissue, reconstruction of the IVS with larger patches, tricuspid valve replacement (TVR), and biventricular pacing were performed.

The primary problem with patch reconstruction is the creation of a defective IVS. As shown in Figure 2D, we set 2 suture lines in the ventricle to sandwich the posterior descending artery and achieved septation by passing the mattress suture toward the outside of the heart through 2 bovine pericardial patches and fixing them with felt strips. It is necessary to place an appropriately sized patch because an excessively large patch might shift to the RV side during the systolic phase, reducing RV volume and function. This can lead to low-output syndrome due to LV dyskinesis, which occurs asynchronously. However, if the patch is too small, strong tension will be applied to the suture, leading to a risk of suture site breakage, possibly resulting in patch leakage or LV rupture after operation.

Another problem is that tricuspid annulus débridement is more likely to require concomitant TVR and pacemaker implantation because of valve malfunction and conduction disturbance. Preventing the recurrence of infection is the most critical issue and should have precedence over the disadvantages of TVR and permanent pacemaker implantation. Implantation of prostheses such as prosthetic valves and pacemaker leads may be at risk for infection recurrence. However, as shown in our case, sufficient débridement of the infected tissue and appropriate reconstruction methods are effective strategies.

In conclusion, we report an extensive operation for MRSA-infected patches collapsing after repair of postinfarction VSR requiring total IVS reconstruction. Our method can achieve a reliable biventricular reconstruction.
